# ReCell Autologous Regeneration Techniques Combined With Traditional Chinese Medicine in the Treatment of Stable Vitiligo: A Retrospective Study

**DOI:** 10.1111/jocd.70973

**Published:** 2026-06-04

**Authors:** Qiao Chen, Xueya Tong, Chen Duan, Wenchao Zhang, Fei Long, Guojing Chang, Xiaohan Hu, Maoying Wei, Zhifei Liu

**Affiliations:** ^1^ Department of Plastic and Aesthetic Surgery Peking Union Medical College Hospital, Chinese Academy of Medical Sciences & Peking Union Medical College Beijing China; ^2^ Department of Dermatology Chengdu Borun Vitiligo Hospital Chengdu China; ^3^ Department of Plastic Surgery The Second Hospital of Hebei Medical Hospital Shijiazhuang China

**Keywords:** Quchong Banjiuju pill, ReCell, repigmentation, stable vitiligo, traditional Chinese medicine

## Abstract

**Background:**

ReCell autologous cell transplantation demonstrates efficacy in vitiligo repigmentation. Traditional Chinese medicine, such as Quchong Banjiuju Pill, may synergize with ReCell to enhance results, though combined protocols remain underexplored.

**Aims:**

This study evaluated the efficacy and safety of ReCell combined with Quchong Banjiuju Pill for stable vitiligo.

**Methods:**

We retrospectively analyzed patients with stable vitiligo who had been treated with ReCell in combination with Quchong Banjiuju Pill for > 6 months. The repigmentation of the lesion areas, the pigmentation grade, and the incidence of adverse reactions were calculated under a Wood's lamp at the 3, 6, 12, and 24 month postoperative follow‐up assessments.

**Results:**

Patients were stratified by lesion location (face/neck, trunk, limbs). Repigmentation rates progressed from 68.08% (3 months) to 85.11% (24 months), with face/neck lesions showing superior outcomes (non‐significant intergroup difference, *p* > 0.05). Two cases developed Köbner phenomenon (trunk/limbs), but no scarring, infections, or uneven pigmentation occurred.

**Conclusions:**

The combination of ReCell and Quchong Banjiuju Pill improved the repigmentation efficacy in patients with stable vitiligo, except for Kobner's phenomenon in 2 cases. No other related adverse reactions were seen in the treatment process. Therefore, the combination of ReCell and Quchong Banjiuju Pill not only improves clinical outcomes but is also safe for use in stable vitiligo.

## 1 Introduction

Vitiligo is a disease in which the lack of melanocytes results in the loss of skin pigment. The clinical manifestations are more obvious in the yellow race, which often brings great distress and psychological pressure to patients. The exact pathogenesis of vitiligo is still unclear [1]. Comprehensive treatment is the main way to treat vitiligo, but through comprehensive treatment, only about 15% of patients have stable and complete color relapse. Traditional surgical treatments include skin graft, micro‐drilling graft, and negative pressure blister epidermal graft. These methods are limited to small lesions, time‐consuming and strenuous, and can also lead to pebble‐like deformity and pigmentation in the donor area, seriously affecting clinical application.

ReCell is an autologous skin cell suspension preparation technology that utilizes trypsin to digest the skin to obtain a suspension with a large number of keratinocytes and melanocytes, which can be used to treat vitiligo with a relatively large area. In recent years, it has been reported that ReCell has achieved a certain curative effect in patients with stable vitiligo, but the effective rate is only 62.68% [2], and its curative effect needs to be improved. At present, there is still a lack of research on ReCell combined drug therapy [3].

Quchong Banjiuju Pill (also called Compound *Vernonia Anthelmintica* Pill) is a traditional Chinese medicine, composed of Vernonia species, made of various herbal medicines, and is an optional medicine for the treatment of vitiligo in the Chinese population. Quchong Banjiuju Pill is a mature Chinese patent medicine approved for marketing by the China National Medical Products Administration, with several decades of clinical application history. Studies have shown that vitiligo can be treated by regulating the immune function of the body, regulating the activity of tyrosinase in the body, and supplementing the content of local trace elements [4]. Clinical research evidence indicates that this pill combined with melanocyte transplantation for the treatment of stable vitiligo exhibits significant advantages: multiple retrospective analyses and clinical observational studies consistently show that patients who received oral pills following autologous epidermal melanocyte transplantation had significantly superior efficacy compared to the control group receiving transplantation surgery alone [4, 5]. The combination therapy significantly improved the excellent and good rate of repigmentation in postoperative lesions, promoted pigment regeneration, and may shorten the time required to achieve ideal repigmentation outcomes [6, 7]. This suggests that Quchong Banjiuju Pill may provide a more favorable “survival and proliferation microenvironment” for transplanted melanocytes [8, 9]. We retrospectively analyzed the long‐term efficacy and safety of ReCell combined with Quchong Banjiuju Pill in the treatment of patients with stable vitiligo.

## 2 Methods

### 2.1 Clinical Patient Selection

In this study, the medical records of all patients with stable vitiligo admitted to the hospital from January 2021 to May 2022 were retrospectively analyzed.

Inclusion criteria: (a) no new lesions over 12 months and no Kobner phenomenon observed; (b) Recell surgery after admission and long‐term use of Qinchong Banjiuju Pill. Exclusion criteria: (a) patients with systemic disease or cicatricial constitution; (b) pregnant women; (c) incomplete medical records. A total of 47 patients were enrolled, including 24 males and 23 females. According to the location of the lesions, the patients were divided into three groups: face and neck group (16 cases), trunk group (excluding genitalia) (16 cases), and limbs group (except finger or toe tip) (15 cases). Age, duration, and sex distributions were comparable in all groups (Table 1).

**TABLE 1:** General information of patients.

	Facial Neck Group (*n* = 16)	Trunk Group (*n* = 16)	Limbs Group (*n* = 15)	*p*
Male, *n* (%)	8 (50.00%)	9 (56.25%)	7 (46.67%)	
Female, *n* (%)	8 (50.00%)	7 (43.75%)	8 (53.33%)	
Age (y)	22.70 ± 7.50 (16–41)	23.1 ± 9.00 (15–46)	21.60 ± 6.80 (17–40)	0.86
Duration (y)	6.80 ± 4.40 (2–16)	5.50 ± 3.90 (1.1–12)	7.20 ± 4.70 (2.4–17.4)	0.52

### 2.2 ReCell Therapy

**FIGURE 1:**
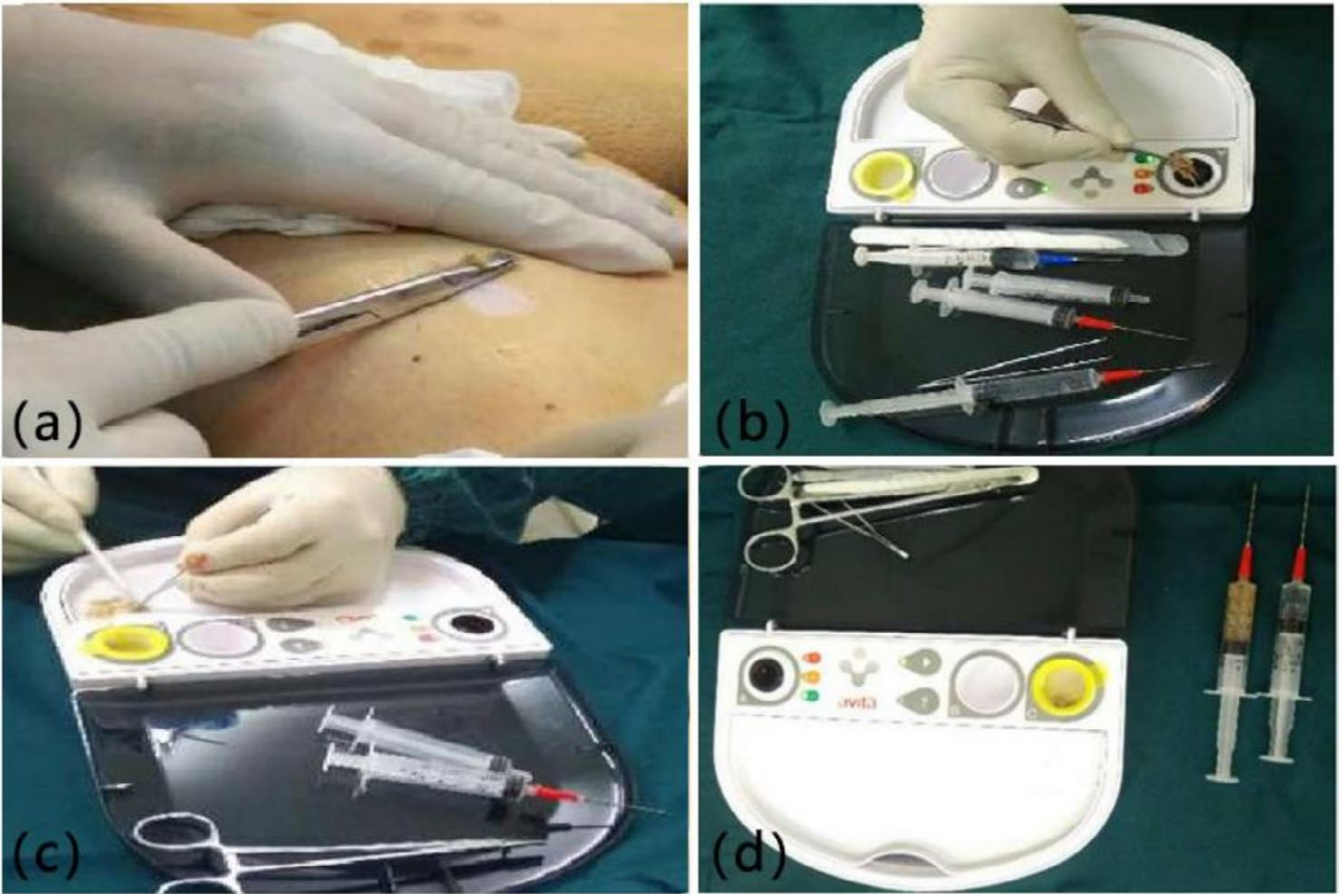
Tissue and cell separation using ReCell. (a) Extraction of skin; (b) Insert the skin into trypsin in the kit; (c) Scraping the cells out of the skin; (d) Preparation of cell suspension.

The donor area is disinfected and a piece of skin 0.15–0.2 mm thick is removed from the non‐damaged area. The skin area of the donor area is about 1/20 to 1/40 of the skin lesion area. The removed skin was placed in a ReCell thermostatic container with trypsin and digested 20–25 min at 37°C. The epidermis is manually separated from the dermis with tweezers after 20–25 min. A cell suspension containing keratinocytes, melanocytes, macrophages, and other components was prepared by scratching the dermal surface of the epidermis with a surgical blade (Figure 1).

### 2.3 Preparation of the Receiving Area

**FIGURE 2:**
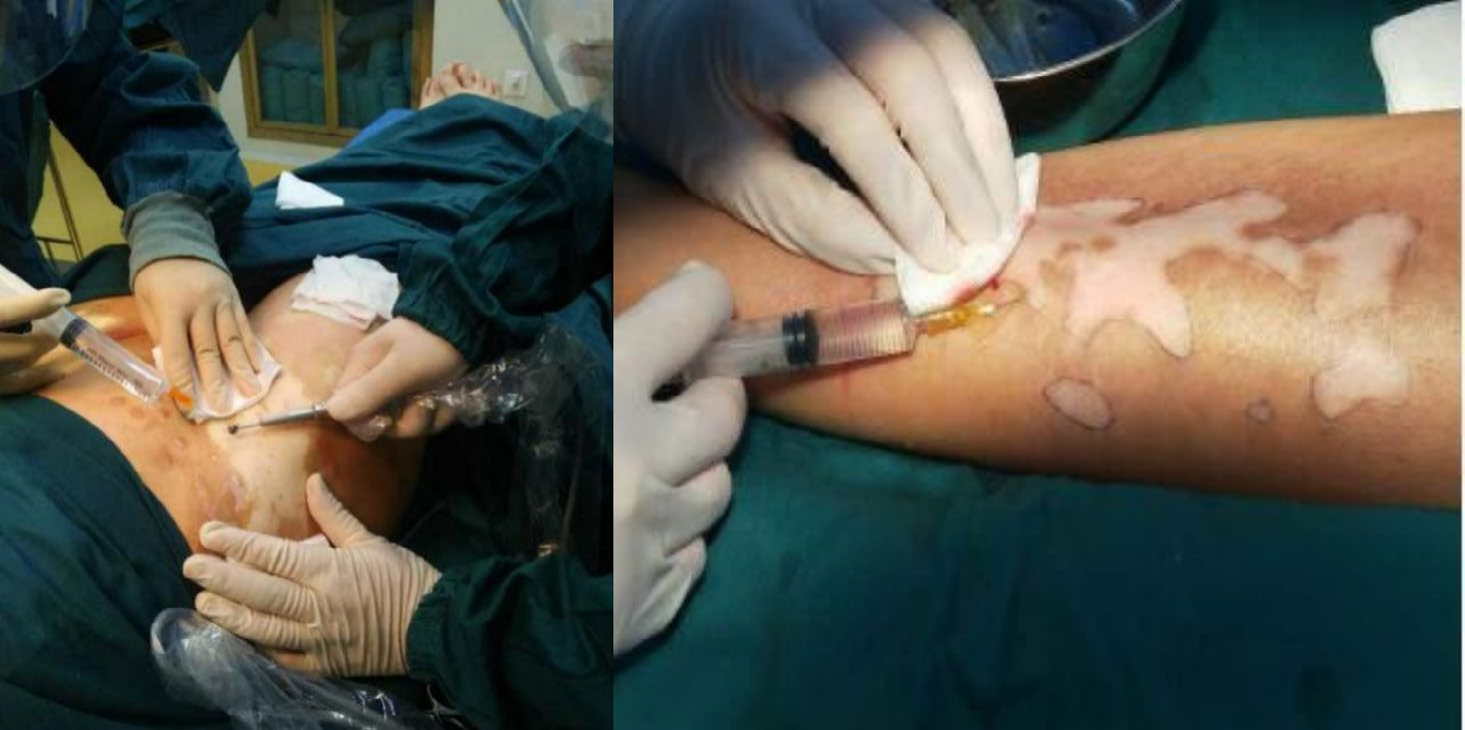
Local anesthesia was administered to the vitiligo recipient site (1% lidocaine without epinephrine).

Local anesthesia was administered to the vitiligo recipient site (1% lidocaine without epinephrine). The site was dermabrased with a micro‐dynamic system to the depth at the cuticle‐dermal junction to the superficial surface of the dermis and removed the epidermis of the white spots (Figure 2).

### 2.4 Cell Suspension Is Sprayed Into the Recipient Site

**FIGURE 3:**
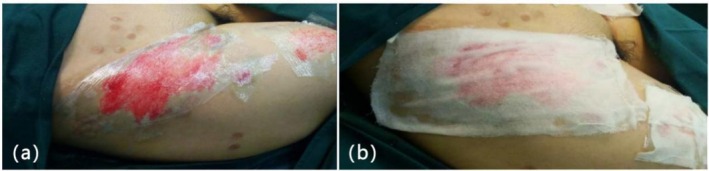
Wound dressing after operation. (a) 3 M Tegaderm I.V. clear dressing cover; (b) Gauze pressure dressing.

The cell suspension was prepared in a ratio of 1:20 to 1:40 and sprayed evenly on the recipient site. It was covered with 3 M Tegaderm I.V. clear dressing, followed by pressure dressing with gauze for 7 days (Figure 3).

### 2.5 Combined Oral Q Drug Treatment

On the day after surgery, the patient was treated by oral administration of Q drug (Z65020018, Kashgar Kunlun Uyghur Pharmaceutical Co. LTD.), 4 tablets each time, 3 times a day. It lasts for 2 years. The pill is made up of insect repellent Turtledove, anachi root, dried ginger, and caryophylla root. The drug has the effects of drying dampness and dispelling wind, relaxing meridian and activating collagalories, activating blood and removing stasis, and increasing skin photosensitivity. It contains trace elements that promote the synthesis of melanin and restore the skin color in the white spots.

### 2.6 Evaluation Indicators

Patients were followed up at 3, 6, 12, and 24 months after surgery. Photographs were taken with a Canon camera (SX600, Japan) under natural light and Wood light during each follow‐up visit. Photographs were taken during each follow‐up and the area size was measured using the grid method. According to the method in reference [10], pigmentation grades were classified as follows: (a) excellent complexing: complexing area ≥ 76%; (b) compound color: compound color area up to 51%–75% of the skin area; (c) medium complexation: complexation area up to the area of skin damage > 26%–50%; (d) compound color difference: compound color area up to skin loss area ≤ 25%. Effective rate = (number of excellent complex‐color cases + number of good complex‐color cases)/total number of cases ×100%; transplantation success is excellent complex‐color + good complex‐color, transplantation failure is medium complex‐color + chromatic aberration. Possible adverse reactions such as keloid or hypertrophic scars, pigmentation, and repigmentation (isomorphic reactions) were recorded to evaluate the safety of this treatment.

### 2.7 Statistical Analysis

Counting data is expressed in frequency/rate (%). SPSS 22.0 was used for χ2 test, and Fisher's exact test was used for data that did not meet the requirements of χ2 test. The significance level was 0.05, and *p* < 0.05 represented a significant difference.

## 3 Results

### 3.1 Efficacy

**FIGURE 4:**
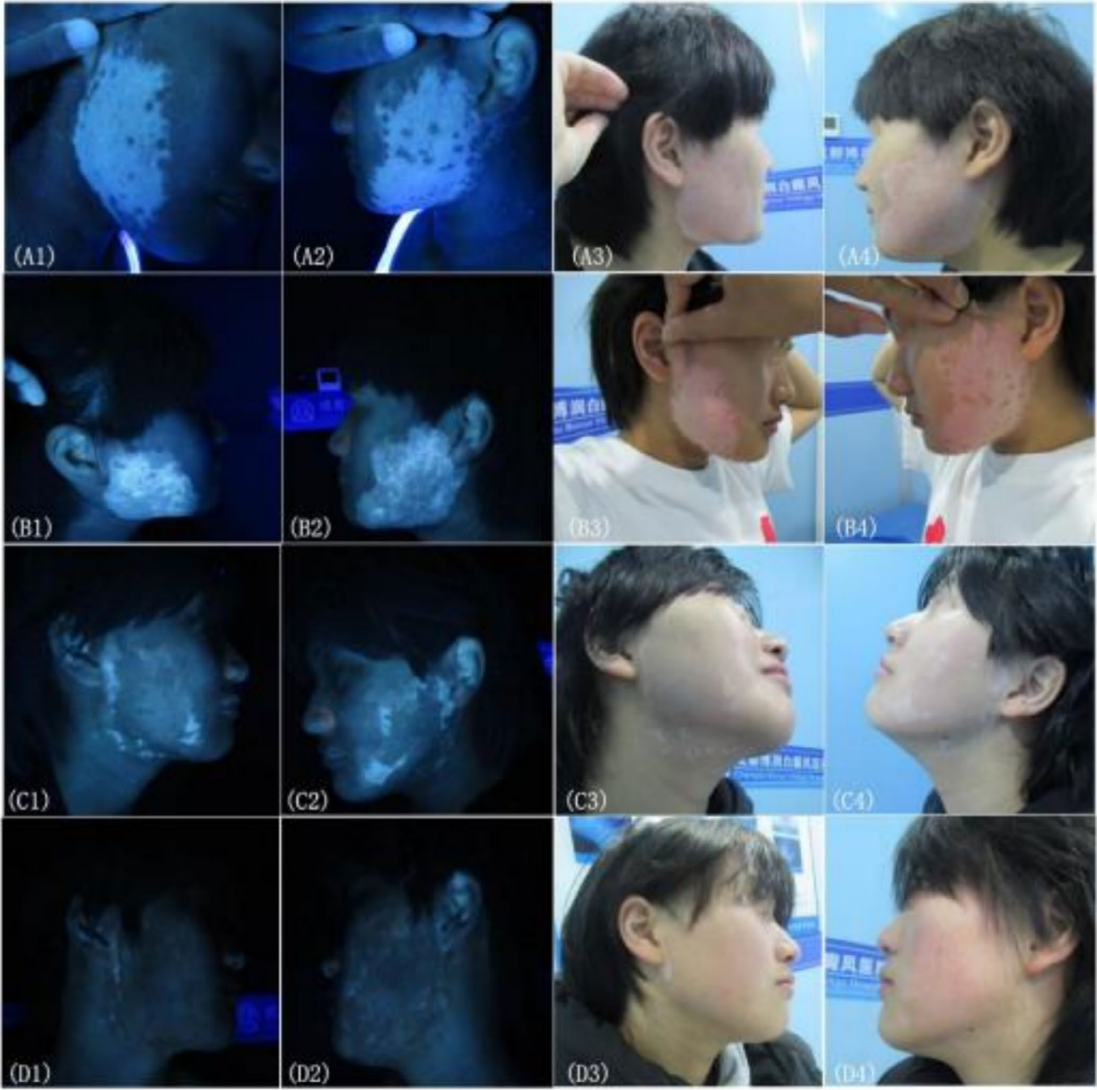
Preoperative and Postoperative photographs of a 16 year‐old patient with vitiligo under natural light (A3, A4, B3, B4, C3, C4, D3, D4) and Wood light (A1, A2, B1, B2, C1, C2, D1, D2). (A1‐A4) before treatment. (B1‐B4) 1 week after treatment. (C1‐C4) 3 months after treatment. (D1‐D4) 6 months after treatment.

**FIGURE 5:**
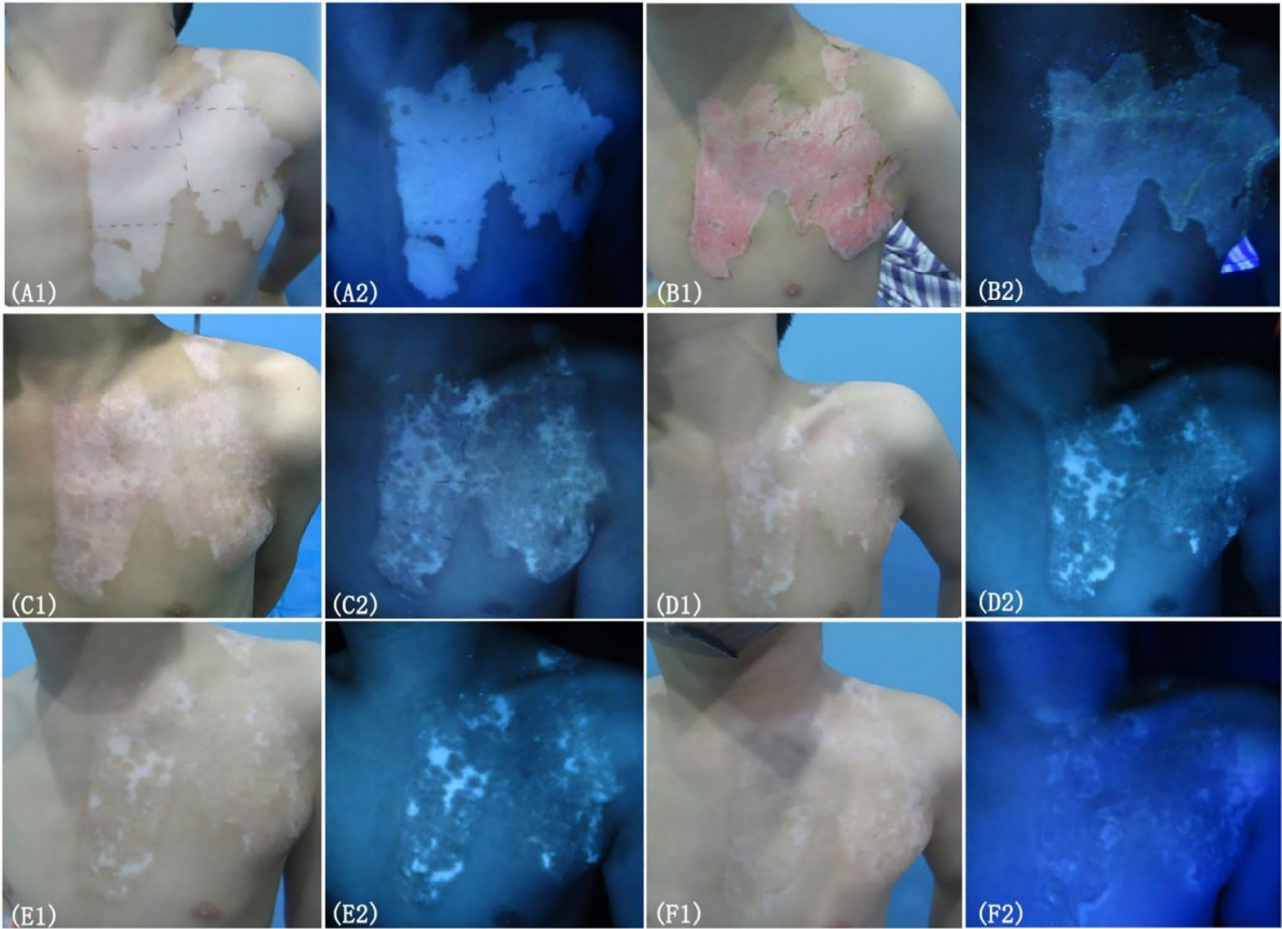
Preoperative and postoperative photographs of an 18 year‐old patient with vitiligo under natural light (A1, B1, C1, D1, E1, F1) and Wood light (A2, B2, C2, D2, E2, F2). (A1–A2) before treatment. (B1–B2) 1 week after treatment. (C1–C2) 3 months after treatment. (D1–D2) 6 months after treatment. (E1–E2) for 12 months after treatment. (F1–F2) 24 months after treatment.

The results showed no significant differences in efficacy or effective rates among the three groups at any follow‐up time point. At 3, 6, 12, and 24 months, the overall efficacy rates were 68.08%, 78.72%, 80.85%, and 85.11%, respectively, with all intergroup comparisons yielding *p* > 0.05 (see Tables 2 and 3 for detailed statistics). Figures 4 and 5 show representative clinical images of two patients before and after treatment. Preoperative and postoperative photographs of patient with vitiligo under natural light and Wood light were taken. The repigmentation outcomes were markedly favorable, demonstrating excellent clinical results.

**TABLE 2:** Efficacy among the Three Groups (3, 6, 12, and 24 Months after Surgery).

Time	Group	Sample Size	Excellent	Good	Medium	Poor	Total Effective Rate
3 months	Facial neck group	16	7 (43.75%)	5 (31.25%)	3 (18.75%)	1 (6.25%)	75%
	Trunk group	16	5 (31.25%)	5 (31.25%)	2 (12.5%)	4 (25%)	62.5%
	Limbs group	15	4 (26.67%)	6 (40%)	1 (6.67%)	4 (26.66%)	66.67%
6 months	Facial neck group	16	11 (68.75%)	3 (18.75%)	1 (6.25%)	1 (6.25%)	87.5%
	Trunk group	16	8 (50%)	5 (31.25%)	1 (6.24%)	2 (12.5%)	81.25%
	Limbs group	15	7 (46.67%)	3 (20%)	2 (13.33%)	3 (20%)	66.67%
12 months	Facial neck group	16	11 (68.75%)	3 (18.75%)	1 (6.25%)	1 (6.25%)	87.5%
	Trunk group	16	10 (62.5%)	4 (25%)	2 (12.5%)	0 (0%)	87.5%
	Limbs group	15	8 (53.34%)	2 (13.33%)	2 (13.33%)	3 (20%)	66.67%
24 months	Facial neck group	16	13 (81.25%)	2 (12.5%)	1 (6.25%)	0 (0%)	93.75%
	Trunk group	16	11 (68.75%)	3 (18.75%)	1 (6.25%)	1 (6.25%)	87.5%
	Limbs group	15	7 (46.67%)	4 (26.66%)	1 (6.66%)	3 (20%)	73.3%

**TABLE 3:** Total Effective Rates at 3, 6, 12, and 24 Months after Operation (*n* (%)).

Time	Group	Sample Size	Excellent	Good	Medium	Total Effective Rate
3 months	57	16 (34.04%)	16 (34.04%)	6 (12.77%)	9 (19.15%)	68.08%
6 months	57	26 (55.32%)	11 (23.40%)	4 (8.51%)	6 (12.77%)	78.72%
12 months	57	29 (61.70%)	9 (19.15%)	5 (10.64%)	4 (8.51%)	80.85%
24 months	57	31 (65.96%)	9 (19.15%)	3 (6.38%)	4 (8.51%)	85.11%

### 3.2 Adverse Events

There were no adverse reactions in the faciocervical treatment group after treatment. Kobner phenomenon was observed in one patient in the trunk group at 12 months and in one patient in the extremities group at 24 months. This eventually leads to the appearance of new white spots.

## 4 Discussion

The pathogenesis of vitiligo is highly complex, involving theories related to autoimmunity [11], genetics, and deficiencies in trace elements [12]. These factors can independently or synergistically cause abnormalities in the local environment of depigmented areas, such as an overactive Th17‐mediated inflammatory response in the local microenvironment of the depigmented areas, with inflammatory cytokines such as IFN‐γ, IL‐6, and IL‐8 causing damage to melanocytes [13]; a reduction in the immunosuppressive function of Treg cells [14], leading to the persistence of autoinflammatory responses and damage to melanocytes; and a significant accumulation of reactive oxygen species in the depigmented areas, inducing oxidative stress and placing epidermal melanocytes in a state of oxidative stress, resulting in cellular damage. Damage to melanocytes, apoptosis or inactivation of pigment cells, leads to the formation of vitiligo [15].

Treatment options for vitiligo are varied. However, despite these approaches, some patients develop treatment‐resistant depigmented patches that often necessitate surgical intervention [15]. Traditional surgical treatments are suitable only for patients with small areas of depigmentation and are time‐consuming and labor‐intensive, with potential complications such as stitch deformities and hyperpigmentation, which limit their widespread application in treatment. Melanocyte transplantation and other cellular transplantation methods are novel surgical techniques for the treatment of vitiligo [16, 17]. Despite their promising therapeutic outcomes, these methods are constrained by high costs, the requirement for specialized laboratories, and issues related to contamination during the culture process, which have not been adequately addressed [18]. Consequently, these limitations restrict the clinical application of such technologies.

In comparison, the ReCell technology, which is a non‐cultured autologous epidermal cell transplantation technique, prepares a cell suspension containing a large number of keratinocytes and melanocytes, promoting repigmentation of vitiligo patches and achieving therapeutic effects [19]. The ReCell technique overcomes the disadvantage of small treatment area associated with tissue transplantation and avoids the high requirements of autologous melanocyte culture transplantation, thus offering greater advantages in clinical practice [20]. However, the use of ReCell alone for the treatment of stable‐phase vitiligo has unsatisfactory efficacy rates [2]. There is also a lack of clinical studies combining Quchong Banjiuju Pill with ReCell. Previous studies have found that oral administration of the Quchong Banjiuju Pill can significantly improve vitiligo, which may be related to the effective components within the pill, such as the coffee acid derivative caffeoylquinic acid [21], and flavonoid substances, bungeiside and bungeichalcone [22]. Bungeiside and bungeichalcone can inhibit the secretion of cytokines such as IL‐4, IgG, and TNF‐*α*, thereby playing a role in regulating humoral immune function. Zhou J et al. found that the ethanol extract of Quchong Banjiuju Pill can promote melanin secretion by enhancing the activity of human primary melanocytes (NHMC), affecting the activity of MITF, a key factor in the expression of tyrosinase, and promoting the phosphorylation level of p38 MAPK, thereby reducing the size of vitiligo patches [23]. Adila Tuerxuntayi used LC–MS/MS and Western Blotting techniques to analyze the extract of Quchong Banjiuju Pill and found that it can increase the secretion of TYR, TRP‐1, TRP‐2, and MITF in mouse B16 cells, thereby promoting the synthesis of tyrosinase within the body [24].

In our previous small sample study involving only 6 patients [25], we observed that among patients with stable non‐segmental vitiligo treated exclusively with ReCell postoperatively over a period of 2 to 8 months (with an average of 6 months), 2 out of 5 patients (33.3%) with non‐segmental vitiligo had more than 80.0% pigment restoration in the treatment area; 3 patients (50.0%) had more than 60% pigment restoration. One patient with generalized lesions (16.7%) showed no significant improvement after treatment. In a subsequent study [2], we found that areas that had repigmented in previous treatments responded better to ReCell than areas that had not repigmented, and the overall efficacy of ReCell treatment at 3 months was: excellent repigmentation in 29.85%, good in 32.83%, moderate in 14.93%, and poor in 22.39%; at 6 months, the overall efficacy was: excellent repigmentation in 54.48%, good in 12.69%, moderate in 9.7%, and poor in 23.13%, all of which were lower than the efficacy observed in the current study where ReCell was combined with the administration of the Quchong Banjiuju Pill. In this study, the efficacy on the face and neck was relatively better compared to other areas. Moreover, the efficacy in all treated areas gradually improved with extended treatment duration. No scarring, infection, or related adverse reactions were observed postoperatively; the repigmentation at the surgical sites was uniform, but two patients experienced recurrence. It was understood that one patient had a burn on the surgical site before the recurrence, and the other had recurrence after trauma, both of which were due to different traumatic factors triggering the Koebner phenomenon, and the recurrence may be closely related to changes in the patient's immune status.

## 5 Limitations

The follow‐up time of this study was long and the effect was clear. However, the sample size is relatively small. As the drugs used are traditional Chinese medicine, the study population is relatively simple, which can be supplemented by multi‐center studies in the future. Our research team has previously published studies demonstrating the efficacy of ReCell technique alone in treating vitiligo [2, 25]. In clinical practice, the combination of ReCell technique with oral medication resulted in superior patient satisfaction and postoperative follow‐up outcomes compared to ReCell technique alone. However, as this study did not establish a parallel control comparing “ReCell technique alone” vs “ReCell technique combined with oral medication,” the validity of the combined treatment results indeed has certain limitations. In the future, we plan to conduct parallel controlled studies under identical enrollment criteria and follow‐up periods to further validate the advantages of the combination therapy.

## 6 Conclusion

The combination of ReCell and the Quchong Banjiuju Pill in the treatment of stable‐phase vitiligo has demonstrated satisfactory clinical efficacy. The ReCell procedure is simple to perform, and no infections or scarring were observed during postoperative follow‐up. Therefore, this treatment protocol holds positive significance for the management of patients with stable‐phase vitiligo.

## Author Contributions

Qiao Chen and Xueya Tong contributed equally to this work. Zhifei Liu conceived and designed the study. Qiao Chen, Xueya Tong were involved in drafting the research proposal and the manuscript. Qiao Chen, Chen Duan, Wenchao Zhang, Fei Long, Guojing Chang, Xiaohan Hu, and Maoying Wei participated in the acquisition of data. Specifically, Chen Duan and Maoying Wei were responsible for patient follow‐up data collection and photographing during follow‐up. Zhifei Liu performed the primary surgical procedures, assisted by Guojing Chang and Xiaohan Hu. Fei Long obtained the ethical committee approval. Qiao Chen and Xueya Tong conducted the analysis and interpretation of data and performed the literature review. All authors critically reviewed the manuscript, read, and approved the final version to be published, and agreed to be accountable for all aspects of the work.

## Funding

This study was funded by Peking Union Medical College Hospital, Department of Plastic and Aesthetic Surgery Consolidated Accumulated Surplus Fund (No. ZC201906165).

## Ethics Statement

This study was conducted in accordance with the principles of the Declaration of Helsinki. The study protocol was reviewed and approved by the PUMCH Institutional Review Board. Written informed consent was obtained from all individual participants included in this study.

## Consent

The authors have obtained written informed consent from the patient/patient's parent/legal guardian for the publication of the patient's photographs in this manuscript. To protect the patient's privacy, identifying features have been concealed in the photographs unless specifically essential for the demonstration of the medical condition and with explicit additional authorization for such use. The consent form is available for review by the journal editor upon request.

## Conflicts of Interest

The authors declare no conflicts of interest.

## Data Availability

The data that support the findings of this study are available on request from the corresponding author. The data are not publicly available due to privacy or ethical restrictions.

## References

[1] F. Diotallevi , H. Gioacchini , E. De Simoni , et al., “Vitiligo, From Pathogenesis to Therapeutic Advances: State of the Art,” International Journal of Molecular Sciences 24, no. 5 (2023): 4910. 36902341 10.3390/ijms24054910 PMC10003418

[2] C. Chen , N. Yu , Z. Liu , et al., “New Pigmentation After Medical Treatment Suggests Increased Efficacy of Dermabrasion and Noncultured Epidermal Cell Suspension Techniques in Stable Vitiligo,” Dermatologic Surgery 47, no. 4 (2021): e142–e145. 33038103 10.1097/DSS.0000000000002820

[3] J. Seneschal and K. Boniface , “Vitiligo: Current Therapies and Future Treatments,” Dermatology Practical & Conceptual 13, no. 4 s2 (2023). 10.5826/dpc.1304S2a313S PMC10824325 38241396

[4] J. N. Teng , Clinical Observation of Melanocyte Transplantation Combined With *Vernonia anthelmintica* Pill in the Treatment of Vitiligo [D] (Shihezi University, 2022).

[5] J. N. Teng , H. J. Wang , and X. J. Kang , “Clinical Efficacy and Influencing Factors of Melanocyte Transplantation Combined With Characteristic Chinese Medicine in the Treatment of Stable Vitiligo,” Chinese Journal of Dermatovenereology 9 (2025).

[6] J. H. Chen , Y. Fan , B. Zhang , et al., “Observation on Efficacy of Compound *Vernonia anthelmintica* Pill Combined With Narrow‐Band UVB in Treatment of Vitiligo,” Inner Mongolia Journal of Traditional Chinese Medicine 35, no. 10 (2016): 8.

[7] Y. E. Tian , J. J. Liu , and K. J. Yang , “Clinical Observation on Comprehensive Treatment of Vitiligo,” Chinese Journal of Ethnomedicine and Ethnopharmacy 20, no. 9 (2011): 90–91.

[8] Y. Reyila , Study on Therapeutic Effect and Mechanism of Compound *Vernonia anthelmintica* on Hydroquinone‐Induced Vitiligo Mouse Model [D] (Xinjiang Medical University, 2020).

[9] Dermatology Branch of China Association of Chinese Medicine , “Expert Consensus on Traditional Chinese Medicine Treatment of Psoriasis (2017),” Chinese Journal of Dermatovenereology of Integrated Traditional and Western Medicine 17, no. 3 (2018): 273–277.

[10] B. Coskun , Y. Saral , and D. Turgut , “Topical 0.05% Clobetasol Propionate Versus 1% Pimecrolimus Ointment in Vitiligo,” European Journal of Dermatology 15, no. 2 (2005): 88–91. 15757818

[11] C. Lyu and Y. Sun , “Immunometabolism in the Pathogenesis of Vitiligo,” Frontiers in Immunology 13 (2022): 1055958. 36439174 10.3389/fimmu.2022.1055958 PMC9684661

[12] H. Z. Marchioro , C. C. Silva De Castro , V. M. Fava , et al., “Update on the Pathogenesis of Vitiligo,” Anais Brasileiros de Dermatologia 97, no. 4 (2022): 478–490. 35643735 10.1016/j.abd.2021.09.008 PMC9263675

[13] P. Manga and N. Choudhury , “The Unfolded Protein and Integrated Stress Response in Melanoma and Vitiligo,” Pigment Cell & Melanoma Research 34, no. 2 (2021): 204–211. 33215847 10.1111/pcmr.12947

[14] K. J. Gellatly , J. P. Strassner , K. Essien , et al., “scRNA‐Seq of Human Vitiligo Reveals Complex Networks of Subclinical Immune Activation and a Role for CCR5 in T(Reg) Function,” Science Translational Medicine 13, no. 610 (2021): eabd8995. 34516831 10.1126/scitranslmed.abd8995 PMC8686160

[15] W. L. Chang and C. H. Ko , “The Role of Oxidative Stress in Vitiligo: An Update on Its Pathogenesis and Therapeutic Implications,” Cells 12, no. 6 (2023): 936. 36980277 10.3390/cells12060936 PMC10047323

[16] M. A. Refat , J. P. Strassner , M. L. Frisoli , et al., “Lesional CD8+ T‐Cell Number Predicts Surgical Outcomes of Melanocyte‐Keratinocyte Transplantation Surgery for Vitiligo,” Journal of Investigative Dermatology 143, no. 11 (2023): 2275–2282.e6. 37478900 10.1016/j.jid.2023.03.1689 PMC11140410

[17] J. Li , X. Zeng , S. Chen , et al., “The Treatment of Refractory Vitiligo With Autologous Cultured Epithelium Grafting: A Real‐World Retrospective Cohort Study,” Stem Cells Translational Medicine 13, no. 5 (2024): 415–424. 38513284 10.1093/stcltm/szae009 PMC11092269

[18] T. Sritanyarat , C. Wongpraparut , N. Jansuwan , et al., “Outcomes of Autologous Non‐Cultured Melanocyte Keratinocyte Transplantation in Vitiligo and Nevus Depigmentosus,” Journal of Dermatological Treatment 33, no. 2 (2022): 935–940. 32643482 10.1080/09546634.2020.1793885

[19] J. H. Holmes , “A Brief History of RECELL and Its Current Indications,” Journal of Burn Care & Research 44, no. Suppl_1 (2023): S48–S49. 36567470 10.1093/jbcr/irad045.060 PMC10185229

[20] S. C. Peirce and G. Carolan‐Rees , “ReCell() Spray‐On Skin System for Treating Skin Loss, Scarring and Depigmentation After Burn Injury: A NICE Medical Technology Guidance,” Applied Health Economics and Health Policy 17, no. 2 (2019): 131–141. 30635844 10.1007/s40258-018-00457-0

[21] L. M. Cai , S. X. Huo , J. Lin , et al., “Chemical Constituents of *Vernonia anthelmintica* (L.) Willd,” Chinese Traditional Patent Medicine 34, no. 11 (2012): 2159–2161.

[22] Y. X. Wang , E. Wang , J. Shang , et al., “Caffeoylquinic Acid Class Chemical Constituents in *Vernonia anthelmintica* ,” China Journal of Chinese Materia Medica 37, no. 11 (2012): 1590–1592. 22993987

[23] J. Zhou , J. Shang , F. Ping , et al., “Alcohol Extract From *Vernonia anthelmintica* (L.) Willd Seed Enhances Melanin Synthesis Through Activation of the p38 MAPK Signaling Pathway in B16F10 Cells and Primary Melanocytes,” Journal of Ethnopharmacology 143, no. 2 (2012): 639–647. 22867636 10.1016/j.jep.2012.07.030

[24] A. Tuerxuntayi , Y. Q. Liu , A. Tulake , et al., “Kaliziri Extract Upregulates Tyrosinase, TRP‐1, TRP‐2 and MITF Expression in Murine B16 Melanoma Cells,” BMC Complementary and Alternative Medicine 14 (2014): 166. 24884952 10.1186/1472-6882-14-166 PMC4091957

[25] A. Zeng , Z. F. Liu , X. J. Wang , et al., “Clinical Efficacy of ReCell Technique in the Treatment of Stable Vitiligo,” Chinese Journal of Medical Aesthetics and Cosmetology 20, no. 6 (2014): 444–446.

